# Early but not late time-restricted eating improves an actigraphy-estimated sleep quality in women with overweight or obesity: secondary analysis of the crossover ChronoFast trial

**DOI:** 10.3389/fnut.2026.1823259

**Published:** 2026-05-26

**Authors:** Beeke Peters, Jennifer Jokisch, Julia Schwarz, Bettina Schuppelius, Andreas F. H. Pfeiffer, Andreas Michalsen, Achim Kramer, Olga Pivovarova-Ramich

**Affiliations:** 1 Department of Molecular Metabolism and Precision Nutrition, German Institute of Human Nutrition Potsdam-Rehbruecke, Nuthetal, Germany; 2 German Center for Diabetes Research (DZD), München-Neuherberg, Germany; 3 Institute of Nutritional Science, University of Potsdam, Nuthetal, Germany; 4 Institute of Agricultural and Nutritional Sciences, Martin Luther University Halle-Wittenberg, Halle (Saale), Germany; 5 Department of Endocrinology and Metabolism, Charité – Universitätsmedizin Berlin, Corporate Member of Freie Universität Berlin and Humboldt-Universität zu Berlin, Berlin, Germany; 6 Department of Internal Medicine and Nature-Based Therapies, Immanuel Hospital Berlin, Berlin, Germany; 7 Institute of Social Medicine, Epidemiology and Health Economics, Charité-Universitätsmedizin Berlin, Corporate Member of Freie Universität Berlin and Humboldt-Universität zu Berlin, Berlin, Germany; 8 Laboratory of Chronobiology, Charité-Universitätsmedizin Berlin, Corporate Member of Freie Universität Berlin and Humboldt-Universität zu Berlin, Berlin, Germany

**Keywords:** chrononutrition, eating timing, hunger, satiety, sleep, time-restricted eating (TRE)

## Abstract

**Background:**

Metabolic disorders are closely linked to sleep disturbances. Time-restricted eating (TRE) can improve metabolic disturbances, but its impact on sleep quality is insufficiently studied and recommendations regarding the eating timing in TRE are pending. Our aim was to investigate the impact of early TRE (eTRE) and late TRE (lTRE) on sleep quality in obesity.

**Methods:**

This is a secondary analysis of the randomized crossover trial, which included 31 women with overweight and obesity. Following a 2–4 week baseline period, participants were assigned to either a two-week eTRE (eating 8 a.m−4 p.m.) or a two-week lTRE (eating 1 p.m.−9 p.m.), separated by a two-week washout phase. Sleep metrics were assessed objectively by blinded actigraphy and subjectively using Pittsburgh Sleep Quality Index (PSQI) and self-report of sleep quality. Hunger and satiety were examined using a Visual Analogue Scale (VAS).

**Results:**

Actigraphy revealed no between-intervention differences in changes in sleep metrics, but improvements were observed within eTRE compared with baseline for sleep efficiency (*p* = 0.047), sleep fragmentation index (SFI) (*p* = 0.029), and awakening length (*p* = 0.043). Individuals with lowest sleep quality at the baseline showed its largest improvements in eTRE. PSQI scores and self-reported sleep quality remained unchanged between and within both interventions. There were no differences in evening hunger and satiety scores between eTRE and lTRE, and no correlations between hunger or satiety and sleep quality.

**Conclusions:**

eTRE, but not lTRE, improved objectively assessed sleep quality, and these changes were not related to hunger or satiety. eTRE may be a more effective strategy for improving wellbeing and sleep-related health outcomes. ClinicalTrials.gov number, NCT04351672 (registered on April 17, 2020).

## Graphical Abstract

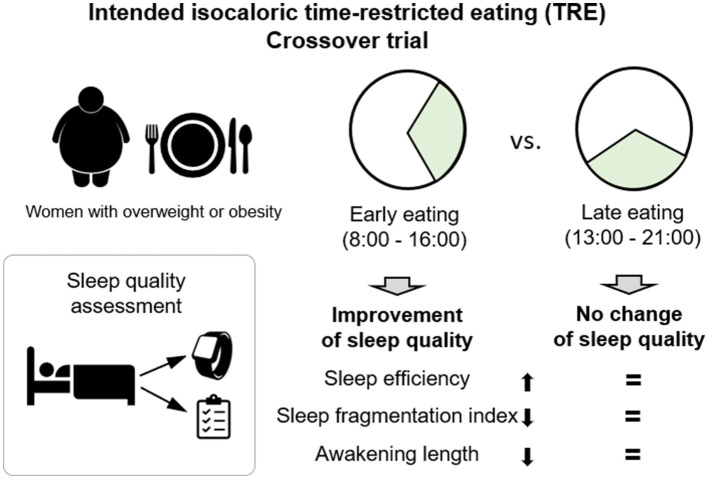

## 1 Introduction

Sleep is important for physical and mental health, quality of life, and wellbeing (1). However, sleep problems are common in the general population (2). Insomnia and chronically reduced sleep duration are associated with hypertension, coronary heart disease, diabetes and obesity (3–5), as well as an increased risk of cognitive impairment (6). Sleep restriction, and possibly sleep fragmentation, are associated with glucose intolerance (7). Conversely, sleep disorders and poor sleep habits are highly prevalent in persons with type 2 diabetes and may be associated with a worse prognosis of sleep-related disorders, such as obstructive sleep apnea or insomnia, in the presence of diabetes (8), suggesting a reciprocal interaction between sleep and metabolic state. However, the causality of this relationship is not sufficiently evaluated (8). Furthermore, sleep deprivation is associated with increases in circulating lipids and weight gain and may promote inflammation – again suggesting a vicious cycle in which risk factors for cardiovascular disease both affect sleep and are affected by sleep (9).

Randomized intervention trials focusing on the treatment of sleep disorders in type 2 diabetes, aimed at improving wellbeing and possibly counteracting diabetes progression, are limited (8, 10). Although pharmacotherapy may offer a quick and convenient approach, once discontinued, it can lead to dependence and a recurrence of sleep disturbances upon discontinuation (11). Therefore, lifestyle-related strategies, including adjustments in eating habits, offer a promising nonpharmacologic way to alleviate sleep disorders (12–14). While improvements in sleep disturbances have been reported with weight loss, changes in dietary composition—such as the use of specific foods or macronutrient distributions—remain insufficiently studied (15). Recently, chrononutrition—i.e., the timing of food intake—has come into greater focus, not least because of its potential role as a link between the modern 24/7 lifestyle and disturbed sleep. Notably, the impact of eating timing on health and sleep quality is largely mediated by circadian clock (16).

According to the two-process model of the sleep regulation, circadian clocks, along with homeostatic regulation, contribute to sleep-wake behavior (17). Circadian clocks synchronize the body's internal processes with the 24-h day-night cycle, influencing sleep-wake patterns, hormone release, and overall alertness. In humans, sleep normally occurs during the biological night and is associated with a decrease in body temperature and the synthesis of melatonin (18). Misalignment of the sleep-wake rhythm with the day-night cycle results in disturbed physiological and hormonal rhythms, with effects at the transcriptional and translational levels (18). Such separation of behavioral cycle (sleep and/or food intake) from the circadian cycle, as occurs with chronic shift work or acute jet lag, is associated with obesity, type 2 diabetes, and other adverse cardiometabolic effects (19, 20). Moreover, a disturbed sleep-wake rhythm can affect eating behavior and appetite regulation (21). Circadian misalignment is tightly associated with decreased sleep efficiency and daytime sleepiness (20, 21). Thus, circadian desynchronization apparently contributes to the bidirectional link between metabolic diseases and poor sleep. Growing knowledge in chronobiology suggests that scheduled eating can act as a zeitgeber and help restore disturbed circadian rhythmicity (22), thereby contributing to improvements in cardiometabolic health.

In fact, time-restricted eating (TRE), a dietary strategy characterized by shortening the daily eating window, has shown beneficial effects on both cardiometabolic outcomes and sleep. However, published research on sleep quality in TRE shows heterogeneous results, which may be partly explained by the use of different objective and subjective measures, as well as heterogeneous study designs. For instance, a number of TRE studies showed no effect on sleep quality assessed by the Pittsburgh Sleep Quality Index (PSQI) in either well-sleeping (PSQI < 5) or poorly sleeping (PSQI >5) subjects (23–27), as summarized in the review of McStay et al. (28). Wilkinson et al. (27) reported a significant increase in sleep quality using the myCircadianClock (mCC) app, but not in PSQI, and no changes in sleep duration and sleep efficiency in a 12-week, 10-h TRE intervention. Only one TRE study observed longer sleep duration after a 16-week, 10-h TRE intervention, but no validated tool was used (29). Regarding sleep latency and sleep efficiency, some TRE trials showed no impact of TRE (23, 26), whereas the study of Lowe et al. (25) reported even a worsening in a 12-week 8-h TRE in individuals with overweight and obesity.

Given heterogeneous data, rigorous studies using objective sleep measures alongside subjective tools are needed to clarify TRE effects on sleep quality and patterns, and general recommendations regarding optimal eating timing are still pending. Therefore, the aim of this study was to compare effects of early (eTRE) vs. late (lTRE) TRE on sleep quality, using both objective and subjective assessments. This study was conducted in terms of the ChronoFast trial (NCT04351672) (30, 31), in which sleep metrics were secondary outcomes.

## 2 Methods

### 2.1 Study design

**Figure 1:**
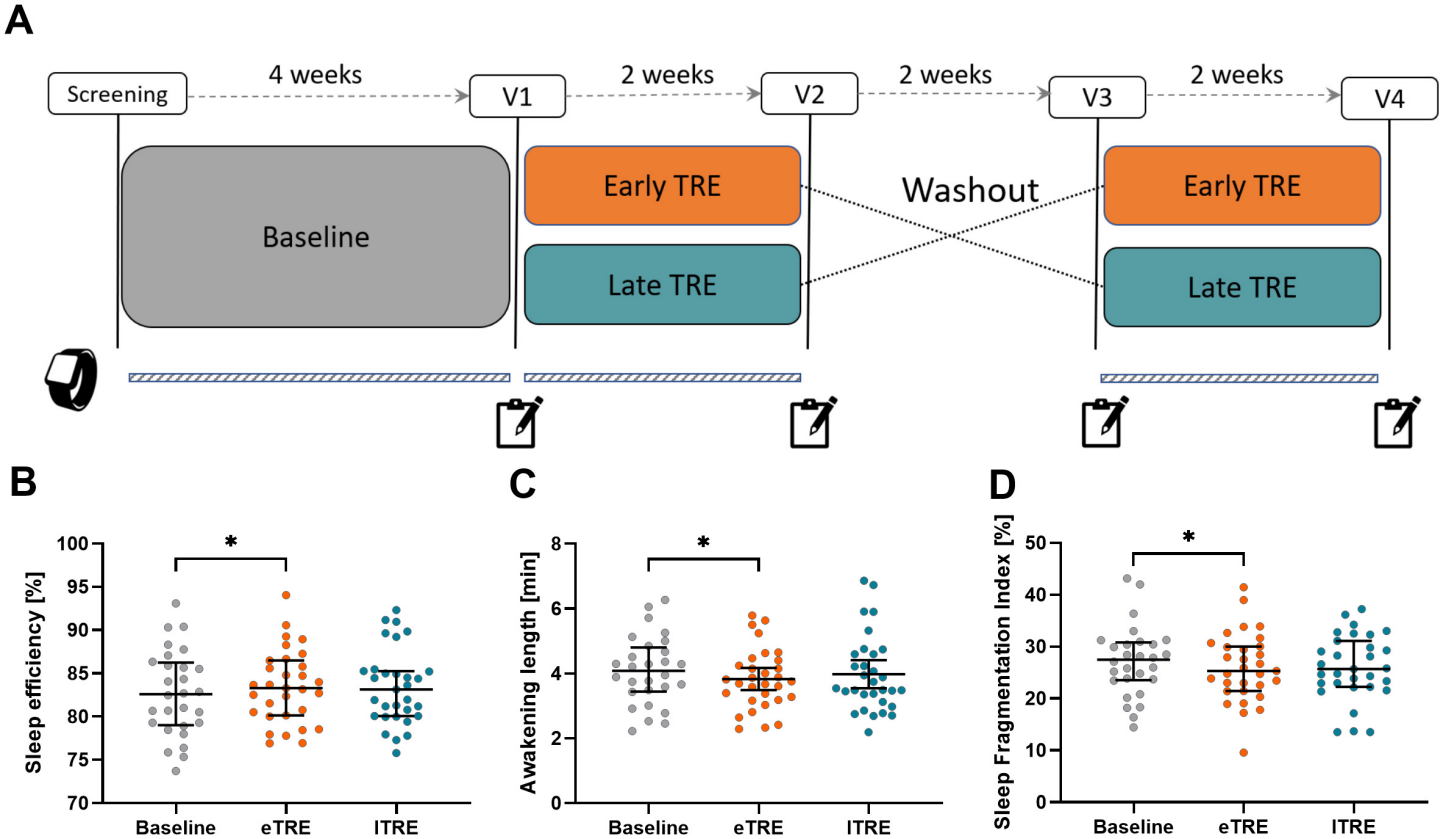
Effects of eTRE and lTRE on objective sleep quality metrics. **(A)** Study design. Baseline (grey), no restrictions of eating timing; early TRE (eTRE, orange), eating window between 8 a.m. and 4 p.m.; late TRE (lTRE, petrol), eating window between 1 p.m. and 9 p.m.; V, visit. Actigraphs were worn for 14 days in the baseline phase and both TRE interventions, whereas questionnaires were collected during every visit (PSQI, self-reported sleep quality score) and at the last day of eTRE and lTRE (hunger and satiety questionnaire). **(B)** Sleep efficiency, **(C)** awakening length, and **(D)** sleep fragmentation index (SFI) in the baseline phase, eTRE and lTRE interventions, as assessed via actigraphy. **(B–D)** Baseline: *n* = 28, eTRE and lTRE: *n* = 31. Data are shown as dot plots with median and IQR. *p < 0.05 by paired Student's *t*-test or Wilcoxon test.

The ChronoFast trial was carried out from March 2020 to December 2021 at the German Institute of Human Nutrition Potsdam-Rehbruecke. ChronoFast was a 10-week randomized, controlled crossover trial that examined the impact of intended isocaloric eTRE vs. lTRE on cardiometabolic health in women with overweight and obesity, without changes in dietary intake and upon nearly unchanged body weight. Participants completed 2-week dietary interventions - eTRE (eating 8 a.m.−4 p.m.) and lTRE (eating 1 p.m.−9 p.m.) - in randomized order. A 2–4-week baseline phase preceded the interventions, and a 2-week washout period separated them (Figure 1A). During the TRE interventions, participants were instructed to eat only within the specified eating windows and not to change energy intake or dietary composition relative to baseline phase. During fasting windows, participants were allowed to consume water, tea, black coffee, and, in small amounts, sugar-free chewing gums or sugar-free soft drinks. The study protocol was approved by the Ethics Committee of the University of Potsdam, Germany (EA No. 8/2019, approved October 25, 2019), conducted in accordance with the Declaration of Helsinki (1975), and registered at ClinicalTrials.gov (NCT04351672 on April 17, 2020). All participants provided written informed consent prior to participation. Detailed study design, samples size calculation for the primary outcome insulin sensitivity, adherence strategies, adverse events, outcome assessments, and data on metabolic and cardiovascular parameters, and internal circadian clocks were published previously (30, 31).

### 2.2 Study cohort

The ChronoFast trial was conducted in 31 female participants aged 18–70 years with a BMI between 25 and 35 kg/m^2^ and without severe sleep disturbances (PSQI < 10). Participants were recruited in the Berlin-Brandenburg region, Germany, via personal interview, posters, flyers, newspaper advertisements, and selected online advertising platforms. Exclusion criteria included factors that could interfere with study variables, such as shift work, travel across time zones, and recent weight change (>5 %). Participants with certain diseases—such as type 1 or type 2 diabetes; severe renal, hepatic, or psychiatric disease—or those taking specified medications were also excluded. A detailed list of exclusion criteria was published previously (30). Participants were randomly allocated to the eTRE-lTRE or lTRE-eTRE study arms based on their BMI and age using the computed minimization method (MinimPy Software) as described (30). Participants' chronotypes were determined using the Munich Chronotype Questionnaire (MCTQ) and the Horne-Östberg Morningness-Eveningness Questionnaire (MEQ) as described (30).

### 2.3 Dietary intake and composition

Weighted food documentation was conducted within the baseline period (for 14 days) and for the whole duration of each TRE intervention (for 14 days) as described (30). In brief, participants were asked to digitally record their food selection, amount, eating times, as well as body weight using the FDDB Extender smartphone app (FDDB Internetportale GmbH, Bremen, Germany) validated previously (32). If participants were not familiar with using a smartphone, they completed handwritten paper-based food records, which were then transferred to the Fddb Extender app by a study assistant. All food records were analyzed for eating times and for energy and macronutrient intake using the FDDB database (Fddb Internetportale GmbH, https://fddb.info/).

### 2.4 Subjective assessment of sleep quality

The PSQI was recorded during screening and at every visit before and after each TRE intervention to assess subjective sleep quality. The PSQI is a widely used questionnaire for the subjective sleep quality assessment, which was developed by Dr. Daniel Buysse, Dr. Charles Reynolds, Dr. Timothy Monk, Dr. Susan Berman, and Dr. David Kupfer at the University of Pittsburgh (33). It comprises 19 items covering sleep duration, sleep latency, sleep efficiency, subjective sleep quality, sleep disturbances, and use of sleep medications, which together yield a global sleep quality score. Higher scores indicate worse sleep quality (33). Translation of the PSQI in German for Germany was performed by Mapi Institute, and a linguistic validation certificate is available on the website of the Center for Sleep and Circadian Science of the University of Pittsburgh (https://www.sleep.pitt.edu/psqi). The German version of PSQI demonstrated high validity and good reliability (34). As an additional subjective measure of the sleep quality in the ChronoFast trial, a school-style grading scale was administered each morning, asking participants to rate the previous night's sleep (1 = “very good” to 5 = “very bad”).

### 2.5 Objective assessment of sleep quality

Objective sleep was assessed using wrist-worn accelerometry (ActiGraph wGT3X-BT; ActiGraph, Pensacola, FL, USA) in the baseline phase (for 14 days) and both TRE interventions (each for 14 days). Participants wore the device continuously on the non-dominant wrist. Removal was required only for swimming or sauna use; any removals were documented with start and end times.

Non-wear logs were used for initial wear-time validation with ActiLife software (version 6.13.4; ActiGraph, Pensacola, FL, USA). Participants recorded bedtime and wake-up time daily while wearing the ActiGraph device. Sleep logs were used to analyze sleep parameters using the Sadeh algorithm as implemented in ActiLife [https://actigraphcorp.my.site.com/].

The following sleep metrics were assessed: total minutes in bed, total sleep time, number and length of awakenings, sleep latency, sleep efficiency, and the sleep fragmentation index (SFI) (35). Total sleep time was defined as the amount of time classified as sleep within the sleep period. Sleep latency was defined as the time the person needs for transition from full wakefulness to sleep, typically after lights out. Sleep efficiency was calculated as total sleep time divided by time in bed, expressed as a percentage The SFI, an indicator of restlessness during the sleep period, was calculated as the sum of the movement index (= number of scored epochs with ≥1 activity counts / total bed time [h] x 100) and fragmentation index (= percentage of 1 min sleep bouts relative to all sleep bouts during the sleep period (or awakenings +1)). For analysis, logged bedtimes and wake times were entered manually by study staff into the ActiLife software as recommended in the software guideline [https://actigraphcorp.my.site.com/support/s/article/Understanding-Latency-in-ActiLife], enabling calculation of sleep latency (= minutes between in-bed time and sleep onset). Days with less than 50% wear time, as well as days when the ActiGraph was issued to or returned by participants, were excluded from analysis.

### 2.6 Hunger and satiety scores

Hunger and satiety were assessed using a questionnaire with four items: (1) desire to eat now, (2) current feeling of hunger, (3) current feeling of satiety, and (4) the amount of food that could be consumed. On the last day of each TRE intervention, participants completed the questionnaire at 8:00 p.m., marking their responses on a 10-cm visual analog scale (VAS; 0 = “not at all” to 10 = “fully applicable”).

### 2.7 Statistical analysis

Statistical analysis was performed using SPSS 25.0 software (IBM, Chicago, IL, USA). Data were presented as mean (SD) unless stated otherwise. Changes within eTRE or lTRE intervention (calculated as a delta of the value after the intervention minus the value before the intervention) and between-intervention differences (calculated as a delta of the change within lTRE minus the change within eTRE) were reported as mean (95% CI). The distribution of the data was determined using the Shapiro–Wilk test. Changes within-intervention and differences between-intervention were expressed as mean difference with 95% confidence interval (CI). Between-intervention (parameter changes in lTRE vs. changes in eTRE) comparisons for anthropometric and sleep quality parameters were assessed by linear mixed models. The model included anthropometric or sleep parameters as dependent variables, treatment (eTRE or lTRE), period (first or second) and residual effect of the first experimental period over the second period (carryover effect) as fixed factors, and subjects as a random factor (30). For sleep quality parameters, the model was adjusted for weight changes which are a potential cofounder. No outcomes showed period or carryover effects. Within-intervention comparisons (values after/during the intervention vs. values before the intervention) and between-intervention (parameter changes in lTRE vs. changes in eTRE) comparisons for all other outcomes were assessed using paired Student's *t*-test for normally distributed data or the Wilcoxon test for non-normally distributed data. For three-group comparisons in tertile analysis, an one-way ANOVA with Tukey's *post hoc* test was applied after the verification of the homogeneity of variances. Correlations were assessed using a Pearson's test. *P* < 0.05 was considered statistically significant in all analyzes. All graphs were generated with GraphPad Prism software, version 10.2.3 (GraphPad Prism Inc, La Jolla, CA, USA).

## 3 Results

### 3.1 Clinical characteristics and sleep timing of participants

A total of 90 individuals were prescreened by telephone by study staff, 36 of prescreened subjects met the eligibility criteria and underwent in-person screening at the examination center. Of these, 31 entered the baseline phase and were randomized: 15 to the eTRE - lTRE sequence and 16 to the lTRE - eTRE sequence. All 31 subjects completed the study and were included in the analyzes (Figure S1). Clinical characteristics of study population at study entry are shown in Table 1. Subjects were 62 (53–65) years old and had a BMI of 30.5 (0.5) kg/m^2^. 18 participants had normal glucose tolerance, whereas 13 had impaired fasting glucose and/or impaired glucose tolerance. 26 participants were postmenopausal and 5 were premenopausal. 18 women had early, 11 - normal, and 2 – late chronotype.

**Table 1:** Baseline characteristics of study population.

Parameter	Value
Females [*n*]	31
Age	62 (53–65)
Chronotype [*n*, early/normal/late]	18/11/2
Weight [kg]	82.5 (8.4)
BMI [kg/m^2^]	30.5 (2.9)
Waist circumference [cm]	99 (9)
Fat mass [%]	34.1 (6.6)
Fasting glucose [mg/dl]	90 (87–96)
HbA1c [%]	5.50 (5.22–5.70)^a^
Glycemic state by OGTT [n, NGT/IFG-IGT]	18/13
Total cholesterol [mmol/L]	5.63 (0.92)
HDL cholesterol [mmol/L]	1.46 (1.33–1.65)
LDL cholesterol [mmol/L]	3.47 (0.82)
Triglycerides [mmol/L]	1.35 (0.62)
Systolic blood pressure [mmHg]	117 (110–135)
Diastolic blood pressure [mmHg]	75 (68–79)
PSQI	5.68 (2.06)

Data are shown as mean (SD) for parameters with normal distribution or median (25th IQR - 75th IQR) for parameters with non-normal distribution (n = 31). eTRE, early time-restricted eating; lTRE, late time-restricted eating; NGT, normal glucose tolerance; IFG, impaired fasting glucose; IGT, impaired glucose tolerance; PSQI, Pittsburgh Sleep Quality Index; BMI, body mass index; SR-sleep quality, self-reported sleep quality. ^a^n = 30.

Adherence to the eTRE and lTRE eating windows was very high (>96 %) in both interventions as described previously (30). Eating time was reduced from 12:06 (1:35) h at baseline to 7:09 (0:32) h during eTRE and to 6:57 (0:50) h during lTRE intervention (Table S2). A small spontaneous weight loss occurred in both interventions (eTRE: −1.08 kg, *p* < 0.001; lTRE: −0.44 kg, *p* = 0.01) with a between-intervention difference of 0.65 kg (*P* = 0.012) (Table S1). Whereas a minimal caloric reduction could not be avoided within eTRE intervention (eTRE: −167 kcal, *p* < 0.001; lTRE: −97 kcal, *p* = 0.06), macronutrient composition remained unchanged in both interventions compared to baseline (Table S2). Participants did not change their habitual daily physical activity during either intervention, as assessed by metabolic equivalent of task (MET) and the proportions of light, moderate and sedentary activity.

Sleep onset and offset at baseline were 23:29 (0:54) h and 07:23 (0:40) h, respectively. During the TRE interventions, sleep onset and offset shifted in parallel with the assigned eating window, resulting in significant between-intervention differences (sleep onset: 9 min, *p* = 0.048; sleep offset: 20 min, *p* = 0.001 for lTRE vs. eTRE) as reported previously (30) and shown in Table S2. However, sleep duration did not change within or between interventions (Table S2). In the present analysis, we focused on sleep quality outcomes across both interventions.

### 3.2 Objective sleep quality

Objective sleep quality metrics were assessed by actigraphy over 14 days in each study period (Table 2, Figures 1B-D). Between eTRE and lTRE interventions, no alterations of these sleep metrics were revealed, and we therefore conducted an additional exploratory analysis of the within-intervention changes. Total minutes in bed, total sleep time, and sleep latency were unchanged between and within TRE interventions (Table 2). Sleep efficiency increased within eTRE (*p* = 0.047) compared to baseline, but not within lTRE, and TRE-induced changes did not differ between interventions (Figure 1B). The awakening length decreased within eTRE (*p* = 0.043), whereas changes within lTRE and between-intervention difference were not significant (Figure 1C). The awakening number was unaffected by TRE (Table 2). The sleep fragmentation index (SFI) declined within eTRE (*p* = 0.029), did not altered within lTRE, and showed no between-intervention difference (Figure 1D).

**Table 2:** Objective and subjective sleep quality metrics in eTRE and lTRE interventions.

Sleep metrics	Baseline	eTRE		lTRE	Change eTRE (95% CI)	*P*-value vs. baseline^*^	Change lTRE (95% CI)	*P*-value vs. baseline^*^	Difference between lTRE vs. eTRE (95% CI)^a^	*P*-value lTRE vs. eTRE changes^**^
Objective actigraphy-estimated metrics			
Total minutes in bed	477 (46)^c^	478 (55)		488 (51)	−1.86 (−14.65–10.94)	0.768^c^	6.75 (−6.78–20.28)	0.315^c^	8.61 (−6.75–23.97)	0.399^c^
Total sleep time	394 (42)^c^	398 (43)		406 (44)	2.80 (−8.08–13.67)	0.602^c^	9.51 (−1.99–21.01)	0.101^c^	6.71 (−6.30–19.72)	0.476^c^
Awakening number	19.1 (5.8)^c^	19.2 (16.4)		19.9 (6.2)	−0.71 (−1.68–0.25)	0.141^c^	−0.13 (−1.44–1.19)	0.845^c^	0.59 (−0.60–1.77)	0.759^c^
Awakening length [min]	4.08 (1.06)^c^	3.83 (0.93)		3.97 (1.18)	−0.22 (−0.43–−0.01)	0.043^c^	−0.05 (−0.36–0.27)	0.466^c^	0.17 (−0.12–0.47)	0.859^c^
Sleep latency [min]	9.11 (5.95)^d^	9.34 (5.77)^b^		7.30 (5.54)^a^	0.65 (−1.29–2.60)	0.469^e^	−1.38 (−3.79–1.02)	0.280^d^	−2.13 (−4.87–0.62)	0.423^e^
Sleep efficiency [%]	82.7 (5.0)^c^	83.5 (4.2)		83.4 (4.4)	1.13 (0.02–2.24)	0.047^c^	0.97 (−0.48–2.42)	0.181^c^	−0.16 (−1.42–1.11)	0.880^c^
Sleep fragmentation index (SFI) [%]	27.1 (6.8)^c^	26.0 (6.6)		25.9 (6.3)	−1.67 (−3.15–−0.18)	0.029^c^	−1.44 (−3.60–0.72)	0.183^c^	0.23 (−1.62–2.08)	0.551^c^
Subjective metrics	
Self–reported sleep quality	2.40 (0.52)^a^	2.34 (0.51)^a^		2.47 (0.46)^b^	−0.07 (−0.20–0.05)	0.247	0.06 (−0.11–0.23)	0.457	0.12 (−0.04–0.28)	0.357
	Before eTRE	After eTRE	Before lTRE	After lTRE	–	–	–	–	–	–
PSQI	5.32 (2.12)	5.45 (2.42)	5.45 (2.16)	5.68 (1.81)	0.13 (−0.47–0.73)	0.662	0.23 (−0.52–0.98)	0.631	0.10 (−0.78–0.97)	0.547

All data is shown as mean (SD). Missing data are due to defective actigraphs and incorrectly or not completed sleep protocols. SFI, Sleep Fragmentation Index; PSQI, Pittsburgh Sleep Quality Index. ^*^Comparison by paired Student's t-test or Wilcoxon test. ^**^Comparison of changes between eTRE and lTRE by the linear mixed model adjusted for body weight changes.^a^n = 30, ^b^n = 29, ^c^n = 28, ^d^n = 27, ^e^n = 26.

**Figure 2:**
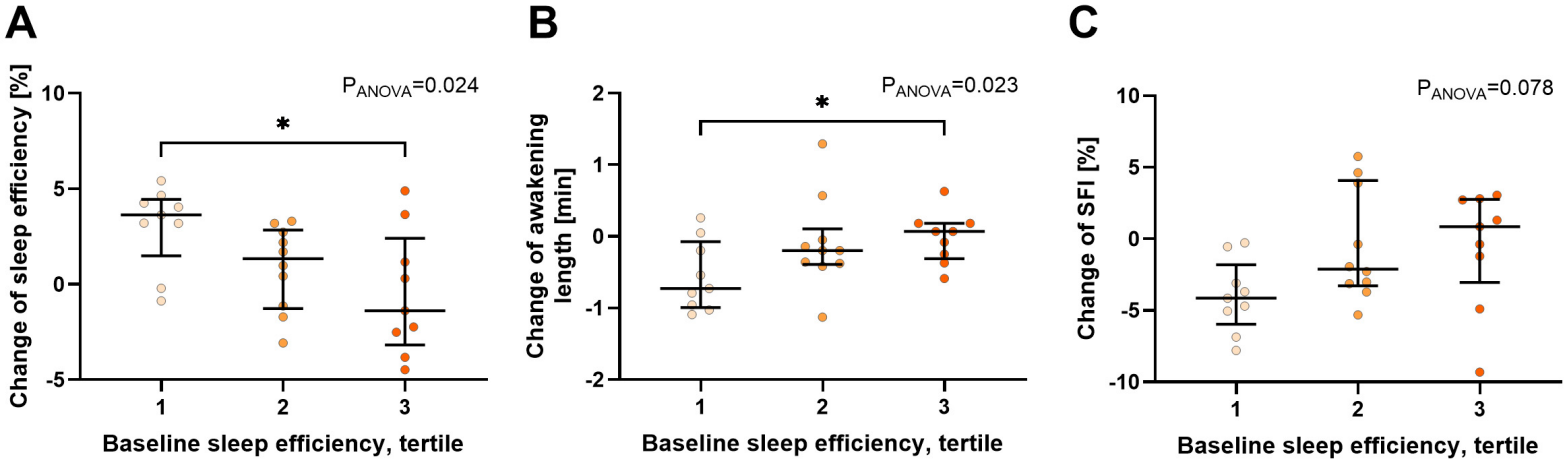
Changes in sleep quality metrics distributed according to sleep efficiency tertiles at the baseline. Changes in **(A)** Sleep efficiency, **(B)** Awakening length, and **(C)** Sleep fragmentation index (SFI) within eTRE are shown. X-axis represents tertiles in sleep efficiency at the baseline, with 1 for lowest values and 3 for highest values. Data are shown as dot plots with median and IQR; *n* = 31. *P*-value by the one-way ANOVA is presented in the right upper corner of the graph. *p < 0.05 between tertiles by Tukey *post hoc* test.

We further examined whether sleep quality and eating timing at baseline predicted subsequent improvements of sleep quality during the intervention. In correlation analysis, baseline sleep efficiency was inversely associated with eTRE-induced changes in sleep efficiency (*r* = −0.523, *p* = 0.004) and awakening length (*r* = −0.575, *p* = 0.001), and positively associated with changes in SFI (*r* = 0.400, *p* = 0.035). In tertile analyzes based on baseline sleep efficiency, participants in the lowest tertile (i.e., with poorest sleep quality) exhibited the greatest increase in sleep efficiency (*p* = 0.022) and the largest decline in awakening length (*p* = 0.017) within eTRE compared to those in the highest tertile, but no difference was observed for SFI (*p* = 0.111) (Figure 2). In contrast, baseline start of eating, end of eating, and eating window duration showed no associations with changes in sleep quality within eTRE (Table S3).

### 3.3 Subjective sleep quality

**Figure 3:**
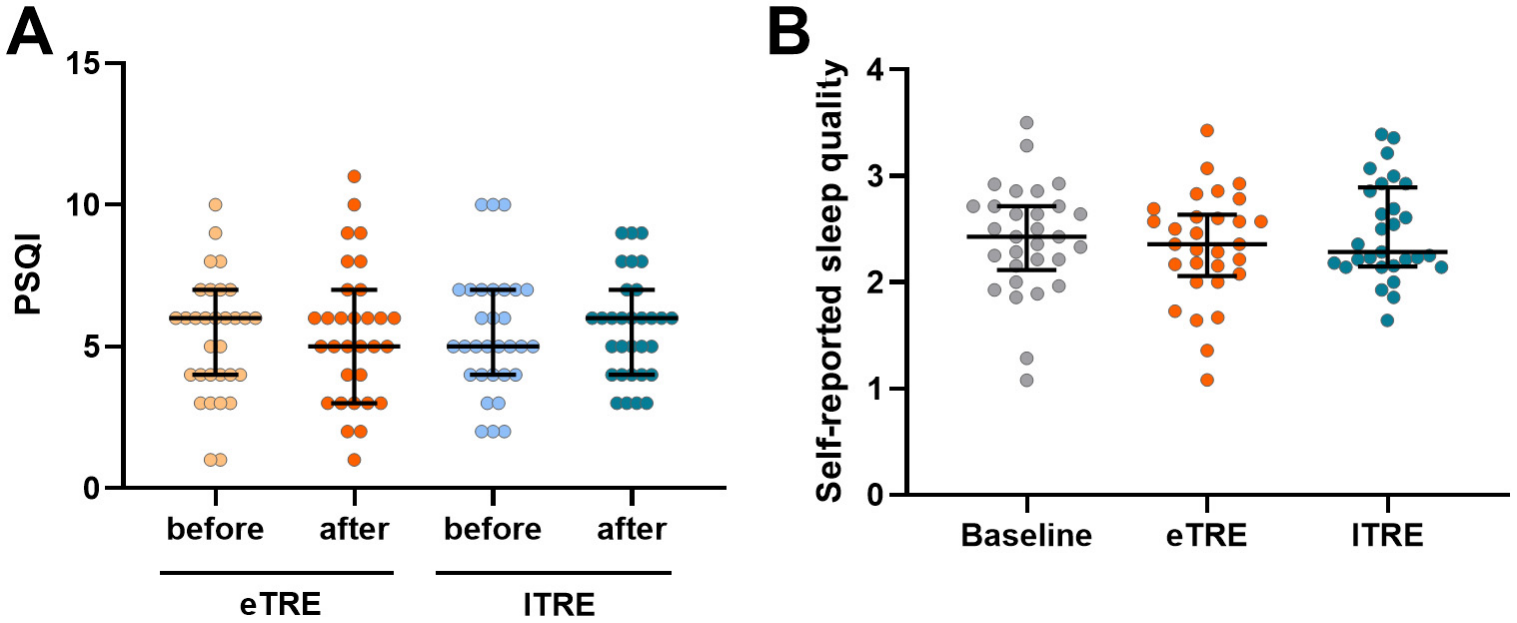
Effects of eTRE and lTRE on subjective sleep quality. **(A)** The Pittsburgh Sleep Quality Index (PSQI) and **(B)** The self-reported sleep quality in the baseline (grey), eTRE (orange) and lTRE (petrol) interventions, as assessed by questionnaires. For PSQI, *n* = 31; for self-reported sleep quality, *n* = 30 for baseline; *n* = 30 for eTRE, *n* = 29 for lTRE. Data are shown as dot plots with median and IQR.

Subjective sleep quality was assessed using the PSQI questionnaire and the self-reported score (Figure 3, Table 2). For PSQI-derived sleep quality, no differences between changes in TRE interventions and no changes in within eTRE and lTRE compared to pre-intervention values were observed (Figure 3A). Self-reported sleep quality was also unchanged between and within TRE interventions (Figure 3B).

### 3.4 Hunger and satiety

Given the restricted eating windows, we hypothesized that feelings of hunger and satiety in the evening might be associated with sleep quality the following night and tested this hypothesis. We observed no differences between eTRE and lTRE in desire to eat, hunger, satiety, or perceived capacity to eat in the evening of the last intervention day, as assessed by VAS (Figure S2). To examine relationships between hunger/satiety and sleep quality, a correlation analysis was conducted. This analysis revealed no associations of hunger and satiety with any assessed sleep quality metric (Table S4).

## 4 Discussion

### 4.1 Early TRE leads to more effective and restful sleep compared to baseline

Our study is the first to investigate the impact of eating timing during TRE on sleep quality by directly comparing early and late eating windows in a crossover trial. The study found no differences between effects of eTRE and lTRE interventions on sleep quality metrics assessed by actigraphy or questionnaires in women with overweight or obesity. Despite the absence of significant between-intervention differences, we cautiously interpreted within-intervention data, considering them as exploratory and hypothesis-generating. In within-intervention comparisons, eTRE - but not lTRE - improved the sleep quality, increasing sleep efficiency, decreasing SFI, and shortening awakening length. Individuals with the lowest sleep quality at baseline showed the greatest improvements in eTRE. Remarkably, these improvements were detected only with using actigraphy as an objective method of the sleep quality assessment, whereas subjective measures (PSQI and a self-reported sleep quality scale) did not capture TRE-induced changes.

The PSQI is a widely used tool for assessing subjective sleep quality (33). A PSQI score < 5 is classified as a good sleep quality (28). In the ChronoFast study, the PSQI at the baseline was 5.68, indicating poor sleep quality at study entry (33). We observed neither positive nor negative change in PSQI-derived sleep quality with either eTRE or lTRE, consistent with most published TRE studies (23–27, 36, 37). For example, a 12-week trial of 8-h eTRE, lTRE, or self-selected TRE found no changes in PSQI scores within any intervention or vs. usual care (36). Similarly, a 6-month trial reported no differences in PSQI-estimated sleep quality within lTRE or compared with either a calorie-restriction group or a usual-diet control (37). Notably, Wilkinson et al. (27) detected improvements in daily, app-based (myCircadianClock) sleep ratings in a 12-week, 10-h TRE intervention, but not in PSQI. Further, Kesztyüs et al. (38) reported VAS-based improvements without PSQI changes. McStay et al. (28) suggested that recall bias may be lower and day-to-day variability better captured with VAS or myCircadianClock-app-based tools than with periodic PSQI administration. Accordingly, we included an additional self-evaluation scale in ChronoFast trial, but again found no effect of eTRE or lTRE on subjective sleep quality. In the review about sleep and TRE by McStay et al. (28) discuss minimal losses of body weight under 2%−3% as a reason for absence of positive effects on sleep quality. In this context McStay et al. (28) further proposed that minimal weight loss (< 2%−3%) may underlie null effects on sleep quality, with benefits more likely after ≥5% loss. However, despite even smaller weight changes in ChronoFast study (~1.2% in eTRE and ~0.6% in lTRE), we observed improvements in objectively assessed sleep quality.

Indeed, in the ChronoFast trial, actigraphy indicated improvements in sleep efficiency and the sleep fragmentation index within eTRE. Consistent with this, awakening length decreased within eTRE, although the awakening number was unchanged. Thus, participants fell asleep more quickly when they awaked at night. In contrast, a recently published TRE study of Lowe et al. (25) reported a worsening of sleep efficiency in TRE with an eating window between 12 a.m. and 8 p.m., which is very similar to the lTRE in the ChronoFast study. Furthermore, a recent randomized trial in women with overweight or obesity reported no significant differences in effects on sleep efficiency and wake after sleep onset when comparing 8-h self-selected TRE, eTRE, lTRE, and usual care combined with a Mediterranean diet (36).

Besides sleep efficiency, the eTRE intervention also led to a significant improvement in SFI. Chung et al. (39) examined how food intake 3 h before bedtime affected sleep quality. The results showed an association with nocturnal awakenings, but no increase in time to fall asleep and no shortening of sleep duration. This suggests that lTRE may have a less positive effect on sleep than eTRE. Therefore, we assume that eTRE led to calmer sleep with fewer interruptions.

Regarding sleep latency, we found no changes within or between eTRE and lTRE. Further, similar to published research (36), no change in sleep duration was observed within both TRE interventions. This result is contradicted only by the study of Gill and Panda (29), who used an unvalidated assessment tool; therefore, their findings should be interpreted with caution (28).

The number of studies investigating the mechanisms by which TRE may influence sleep quality is limited (40), and interpretation is complicated by heterogeneous study designs. Our data on changes on sleep onset and offset (Table S2 and (30)) support the hypothesis that earlier eating windows in eTRE may improve sleep by advancing sleep timing (41). Early eating would better align eating timing with circadian rhythms, in particularly by reducing the overlap between the evening melatonin peak and postprandial insulin secretion, thereby improving glycemic control (42–44). Compared with late eating, early eating might also influence hunger and satiety and, in turn, affect sleep, as discussed below.

### 4.2 Improved sleep metrics are independent from hunger and satiety

A recently proposed hypothesis suggests that insufficient sleep promotes increased energy intake via eating in the absence of hunger (45). Consequently, longer wakefulness provides more opportunities to eat (45). Based on this, and on research showing interactions between dietary modifications and sleep as well as sleep restriction–induced changes in appetite (46), we expected to observe accompanying changes in hunger/satiety due to TRE in the ChronoFast study.

Indeed, a recently published crossover study evaluating lTRE as a potential promoter of positive energy balance reported increased self-reported hunger along with hormonal changes (46). Specifically, the authors reported decreased wake-time 24-h leptin levels and a higher (acetylated) ghrelin:leptin ratio, consistent with greater hunger after the lTRE diet. However, these hunger changes did not translate into differences in total sleep time, sleep efficiency, or wake time (46). In line with a potential advantage of earlier intake, another crossover study found lower hunger when participants consumed most of their daily calories in the morning vs. the evening, supporting the hypothesis that early eating could improve adherence to weight-loss diets (47).

Unexpectedly, in our study, we observed no differences between eTRE and lTRE in evening VAS ratings in desire to eat, hunger, satiety, or perceived capacity to eat, and no associations of hunger and satiety with sleep quality. The discrepancy with prior studies might be explained by nearly isocaloric energy intake and unchanged dietary composition – factors that are often not carefully controlled in TRE trials. Indeed, participants were instructed to replicate their baseline diets and only reduce the daily eating window. Had intake been *ad libitum*, hunger and satiety outcomes might have differed. In particular, people tend to consume more energy-dense snacks and generally more protein and fat in the evening compared to the morning (48, 49), which could modulate hunger/satiety in early and late TRE as described in previous research. Overall, the improved objective sleep quality observed during eTRE in our study is unlikely to be mediated by changes in evening hunger or satiety.

### 4.3 Limitations

Our analysis of TRE effects on sleep quality has some limitations. First, this study included only women, the majority of whom were postmenopausal, limiting generalizability to men and younger populations. Second, only two participants had late chronotypes; while this increased sample homogeneity, it further constrains external validity. Third, individuals with severe sleep disturbances (PSQI ≥10) were excluded because circadian rhythmicity was one of main outcomes of the study (30). Our data showed that TRE could be even more beneficial for individuals with a bad sleep quality, although this effect was not confirmed in a previous study (23). Notably, the exploratory tertile analysis divided the participants according to their baseline sleep efficiency might be also underpowered and warrants further investigation. Fourth, daytime sleep (napping), which may influence nocturnal sleep quality, was not assessed. Moreover, because sleep timing was logged manually, recall bias cannot be ruled out if entries were not made precisely at bedtime. Finally, 2-week intervention phases were relatively short, and the adherence may tend to decline over time in longer trials. Further, individuals who enroll in lifestyle intervention studies are often more motivated than general population. In this context, it is necessary to show whether the effects persist during long-term interventions and under real-life conditions.

## 5 Conclusion

Our study found that, in women with overweight or obesity, an earlier eating window during TRE - but not a later one - improved actigraphy-assessed sleep quality, independent of evening hunger or satiety. Early TRE may therefore be a more effective strategy to enhance wellbeing and sleep-related metabolic health outcomes.

## Data Availability

The raw data supporting the conclusions of this article will be made available by the authors, without undue reservation.
