# Supplementary material for: Early but not late time-restricted eating improves an actigraphy-estimated sleep quality in women with overweight or obesity: secondary analysis of the crossover ChronoFast trial
Source: Front Nutr. 2026 May 26;13:1823259. doi: 10.3389/fnut.2026.1823259 (PMC13246623; doi:10.3389/fnut.2026.1823259)
Supplement: Supplementary file 1 [file Supplementary_File_1.docx]

Supplementary Material

# Peters B, Jokisch J, Schwarz J et al. Early but not late time-restricted eating improves an actigraphy-estimated sleep quality in women with overweight or obesity: secondary analysis of the crossover ChronoFast trial

# Supplementary Tables

**Table S1. Anthropometric parameters in eTRE and lTRE interventions.**

| **Charac**  **teristics** | **Before eTRE** | **After eTRE** | **Change eTRE**  **after – before**  **(95% CI)** | **P*** |  | **Before lTRE** | **After lTRE** | **Change lTRE**  **After – before**  **(95% CI)** | **P*** |  | **Difference between lTRE vs. eTRE (95% CI)^a^** | **P**** |
| --- | --- | --- | --- | --- | --- | --- | --- | --- | --- | --- | --- | --- |
| BMI (kg/m^2^) | 30.4 (2.9) | 30.0 (2.8) | -0.45 (-0.56 to -0.33) | **5.4x10^-9^** |  | 30.3 (2.8) | 30.2 (2.9) | -0.12 (-0.21 to -0.03) | **0.01** |  | 0.33 (0.21 to 0.44) | **6.8x10^-4^** |
| Weight (kg) | 82.3 (8.4) | 81.3 (8.0) | -1.08 (-1.40 to -0.77) | **9.1x10^-8^** |  | 82.0 (8.1) | 81.5 (8.1) | -0.44 (-0.74 to -0.13) | **0.01** |  | 0.65 (0.27 to 1.03) | **0.012** |
| Body fat (%) | 41.7 (0.7)^a^ | 41.5 (0.7)^a^ | -0.24 (-0.81 to 0.32)^a^ | 0.44^a^ |  | 41.5 (0.8)^b^ | 41.7 (0.7)^b^ | 0.18 (-0.35 to 0.70)^b^ | 0.47^b^ |  | 0.42 (-0.18 to 1.02) | 0.82 |
| Waist circumference (cm) | 99.1 (9.5) | 98.8 (8.2) | -0.36 (-1.65 to 0.92) | 0.57 |  | 98.9 (8.2) | 99.3 (8.3) | 0.48 (-0.97 to 1.92) | 0.51 |  | 0.84 (-1.21 to 2.89) | 0.40^c^ |

Data are shown as mean (SD) or mean (95% CI).

* Comparison by paired Student's t-test or Wilcoxon test

** Comparison of changes between eTRE and lTRE by the linear mixed model

^a^ n = 30

^b^ n = 28

^c^ showed significant period effect

**Table S2. Eating and sleeping behavior in eTRE and lTRE interventions.**

| **Eating and sleeping parameters** | **Baseline** | **eTRE** | **lTRE** | **Change eTRE (95% CI)** | **P-value vs. baseline*** | **Change lTRE**  **(95% CI)** | **P-value vs. baseline*** | **Difference between lTRE vs. eTRE (95% CI)^a^** | **P-value**  **lTRE vs. eTRE changes*** |
| --- | --- | --- | --- | --- | --- | --- | --- | --- | --- |
| **Eating behavior** | |  |  |  |  |  |  |  |  |
| Start of eating [hh:mm] | 8:30 (1:03) | 8:31 (0:22) | 13:09 (0:18) | 0:00 (-0:20 to 0:22) | 0.939 | 4:38 (4:14 to 5:02) | **1.2x10^-6^** | 4.37 (4:30 to 4:45) | **1.6x10^-35^** |
| End of eating [hh:mm] | 20:38 (1:11) | 15:42 (0:20) | 20:06 (0:39) | -4:56 (-5:23 to -4:29) | **3.2x10^-20^** | -0:32 (-1:00 to -0:03) | **0.028** | 4:24 (4:12 to 4:36) | **4.2x10^-29^** |
| Eating duration [hh:mm] | 12:06 (1:35) | 7:09 (0:32) | 6:57 (0:50) | -4.57 (-5:32 to -4:21) | **1.2x10^-6^** | -5:08 (-5:47 to -4:29) | **1.2x10^-6^** | -0.11 (-0:24 to 0:01) | 0.110 |
| Energy intake [kcal] **^b^** | 2011 (303) | 1835 (262) | 1916 (383) | -167 (-249 to -86) | **2.4x10^-4^** | -97 (-193 to -2) | 0.06 | 72 (-15 to 159) | 0.10 |
| Protein intake [EN%] **^b^** | 15.5 (2.0) | 15.1 (1.9) | 15.4 (2.1) | -0.4 (-1.1 to 0.3) | 0.27 | **-**0.01 (-0.7 to 0.7) | 0.97 | 0.4 (-0.3 to 1.1) | 0.29 |
| Carbohydrate intake [EN%] **^b^** | 42.9 (39.3 – 46.2) | 43.8 (41.3 – 46.2) | 43.3 (40.1 – 45.6) | 0.2 (-1.5 to 2.0) | 0.78 | -0.7 (-2.2 to 0.9) | 0.38 | -0.9 (-2.4 to 0.5) | 0.23 |
| Fat intake [EN%] **^b^** | 39.8 (36.4 – 42.0) | 39.4 (36.4 – 42.7) | 39.4 (37.7 – 42.9) | 0.1 (-1.3 to 1.5) | 0.86^b^ | 0.8 (-0.6 to 2.3) | 0.25^b^ | 0.7 (-0.6 to 2.0) | 0.28 |
| **Sleep timing** |  |  |  |  |  |  |  |  |  |
| Sleep onset | 23:29 (0:54)^a^ | 23:20 (0:51) | 23:29 (0:51) | -0:09 (-0:19 to 0:01) | 0.08^a^ | 0:00 (-0:07 to 0:08) | 0.95^a^ | 0:09 (0:00 to 0:18) | **0.048**^a^ |
| Sleep offset | 07:23 (0:40)^a^ | 07:10 (0:34) | 07:31 (0.43) | -0:12 (-0:23 to -0:01) | **0.02^a^** | 0:08 (-0.00 to 0:17) | 0.06^a^ | 0:20 (0:09 to 0.32) | **0.001**^a^ |
| Sleep duration | 7:53 (0:51)^a^ | 7:49 (0:55) | 8:01 (0:51) | -0:03 (-0.15 to 0:08) | 0.58^a^ | 0:08 (-0.04 to 0.20) | 0.18^a^ | 0:11 (-0:02 to 0:25) | 0.101 |
| **Physical activity** | |  |  |  |  |  |  |  |  |
| MET | 1.59 (0:15)^b^ | 1.56 (0.14) | 1.57 (0:12) | -0.02 (-0.05 to 0.01) | 0.19^b^ | -0.01 (-0.04 to 0.02) | 0.52 | 0.01 (-0.02 to 0.04) | 0.54^b^ |

Data are shown as mean (SD) for parameters with normal distribution or median (25th IQR - 75th IQR) for parameters with non-normal distribution. Changes are reported as mean (95% CI). Abbreviations: kcal, kilo calories; EN%, energy percent; MET, metabolic equivalent of task.

* Comparison by paired Student's t-test or Wilcoxon test. ^a^ n = 30, ^b^ n = 29, ^c^ n = 28

**Table S3. Correlations of eating timing at the baseline with changes in sleep quality metrics.**

|  |  | **Change of sleep metrics in eTRE** | | |
| --- | --- | --- | --- | --- |
|  |  | **Sleep efficiency** | **Awakening length** | **Sleep fragmentation index (SFI)** |
| Start of eating | r | -0.064 | -0.209 | -0.002 |
|  | (p) | (0.747) | (0.286) | (0.991) |
| End of eating | r | 0.018 | -0.277 | 0.088 |
|  | (p) | (0.928) | (0.153) | (0.657) |
| Eating duration | r | 0.041 | -0.048 | 0.127 |
|  | (p) | (0.837) | (0.808) | (0.519) |

Abbreviations: r, Pearson correlation coefficient; SFI, Sleep Fragmentation Index.

**Table S4. Correlations of actigraphy-estimated sleep quality metrics with hunger and satiety parameters.**

|  |  | **eTRE** | | **lTRE** | |
| --- | --- | --- | --- | --- | --- |
|  |  | **Hunger** | **Satiety** | **Hunger** | **Satiety** |
| Total minutes in bed | r | 0.212 | -0.123 | 0.225 | -0.273 |
|  | (p) | (0.269) | (0.524) | (0.249) | (0.152) |
| Total sleep time | r | 0.239 | -0.024 | 0.294 | -0.311 |
|  | (p) | (0.213) | (0.903) | (0.128) | (0.101) |
| Awakening number | r | -0.060 | 0.049 | 0.044 | -0.136 |
|  | (p) | (0.756) | (0.799) | (0.824) | (0.482) |
| Awakening length | r | 0.096 | -0.352 | -0.023 | 0.066 |
|  | (p) | (0.620) | (0.061) | (0.909) | (0.735) |
| Sleep latency | r | 0.165 | -0.176 | -0.020 | 0.041 |
|  | (p) | (0.411) | (0.381) | (0.922) | (0.838) |
| Sleep efficiency | r | -0.006 | 0.222 | 0.178 | -0.083 |
|  | (p) | (0.973) | (0.246) | (0.366) | (0.668) |
| Sleep fragmentation index (SFI) | r | 0.103 | -0.005 | -0.069 | 0.022 |
|  | (p) | (0.595) | (0.978) | (0.727) | (0.909) |
| Self-reported sleep quality | r | 0.174 | 0.224 | 0.256 | -0.220 |
|  | (p) | (0.376) | (0.252) | (0.206) | (0.270) |
| PSQI | r | -0.030 | -0.181 | 0.102 | 0.043 |
|  | (p) | (0.876) | (0.346) | (0.605) | (0.825) |

Abbreviations: r, Pearson correlation coefficient; SFI, Sleep Fragmentation Index.

# Supplementary Figures

**
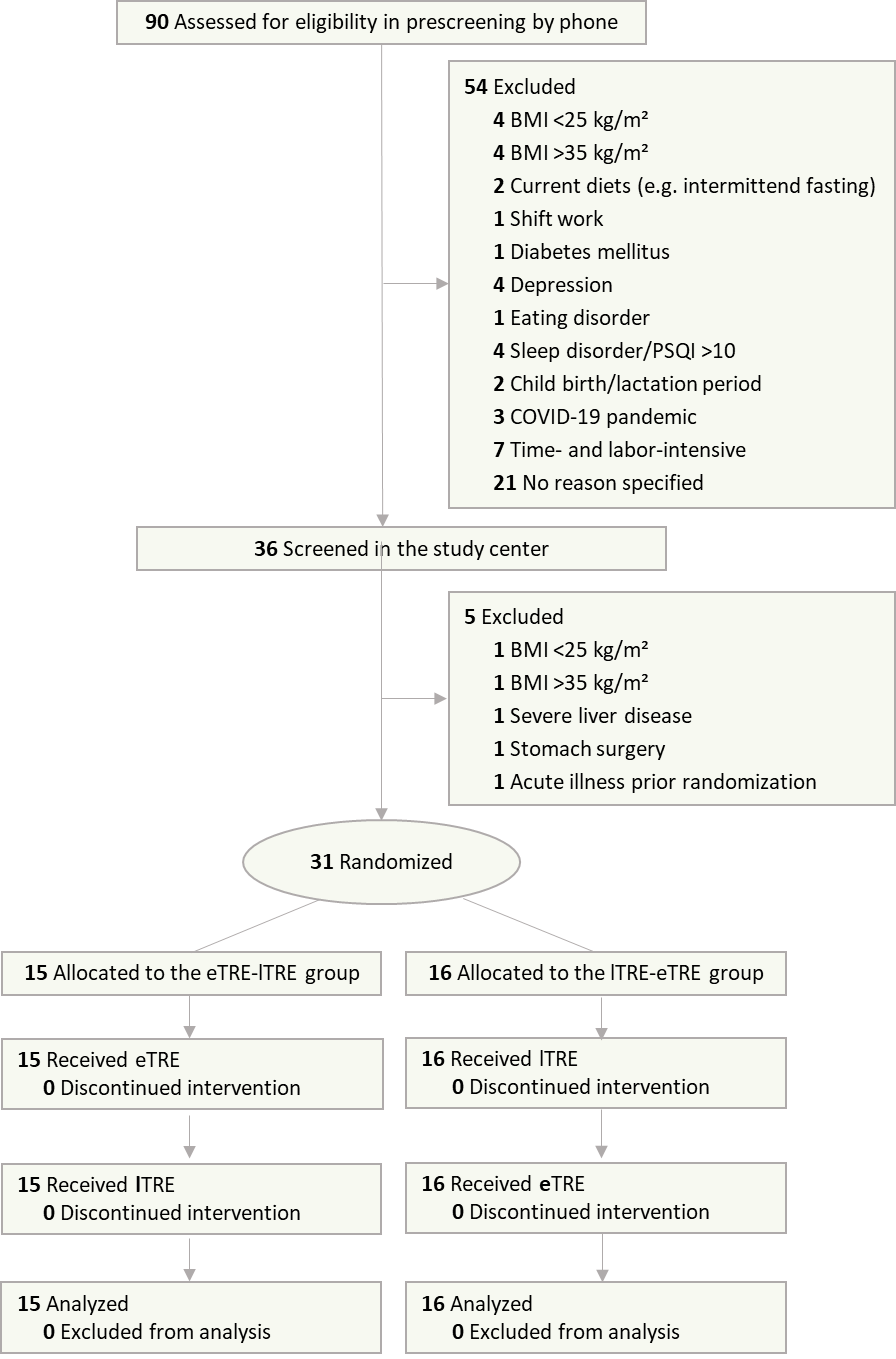
**

**Figure S1. Participant Flow Diagram.**

eTRE indicates early time-restricted eating intervention; lTRE. late time-restricted eating intervention


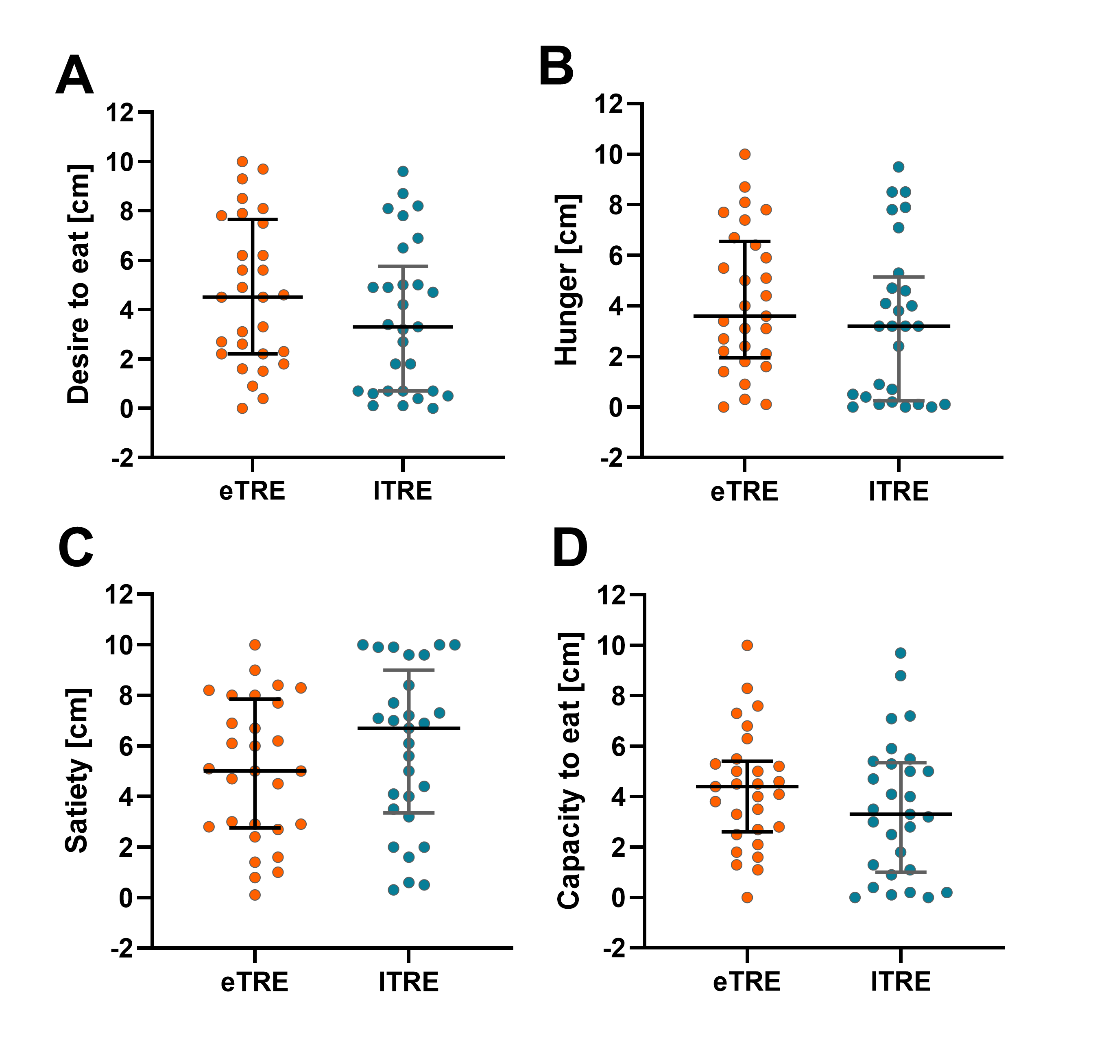


**Figure S2. Hunger and satiety scores in eTRE and lTRE interventions.** (A) Desire to eat, (B) feeling of hunger, (C) feeling of satiety, and (D) capacity to eat in the eTRE (orange) and lTRE (petrol) interventions, as assessed on the last day of each intervention in the evening at 8 p.m., by a visual analogue scale (VAS). (A, C, D) eTRE and lTRE: n = 29; (B) eTRE: n=29, lTRE: n = 28. Data are shown as dot plots with median and IQR. *p < 0.05 by paired Student's t-test or Wilcoxon test.
